# The Application Value of the Central Lymph Node Metastasis Risk Assessment Model in Papillary Thyroid Microcarcinoma of Stage cN0: A Study of 828 Patients

**DOI:** 10.3389/fendo.2022.843573

**Published:** 2022-03-10

**Authors:** Jinqiu Wang, Xianneng Sheng, Yongping Dai, Jiabo Zhang, Lihua Song, Yu Guo

**Affiliations:** ^1^ Department of Thyroid and Breast Surgery, Ningbo First Hospital, Ningbo, China; ^2^ School of Medicine, Ningbo University, Ningbo, China

**Keywords:** papillary thyroid microcarcinoma, central lymph node metastasis, central lymph node dissection, risk factors, surgery

## Abstract

**Background:**

The aim of this study is to build a risk assessment system for central lymph node metastasis (CLNM) in papillary thyroid microcarcinoma (PTMC) of stage cN0 and to explore its application value in clinical practice.

**Methods:**

A total of 500 patients with PTMC who underwent thyroid operation from 2013 to 2015 in Ningbo First Hospital were selected as the model group. Independent risk factors related to CLNM in PTMC were analyzed and determined, and a risk assessment system for CLNM was preliminarily established. Furthermore, the clinicopathological data from 328 PTMC patients with the same conditions as the model group from 2016 to 2017 were further collected as the validation group to verify the diagnostic value of the risk assessment system.

**Results:**

The risk assessment system was based on the score rating (score ≤ 5 was classified as low risk, 6–8 was classified as medium risk, and ≥9 was classified as high-risk). The area under the receiver operating characteristic curve (ROC) was 0.687 (95% CI: 0.635–0.783). According to the risk assessment system, 328 PTMC patients in the validation group were scored. Among the low-risk group, the moderate-risk group, and the high-group, 96.8%, 58.1%, and 43.2% were the CLNM (-) patients, and 3.1%, 41.9%, and 65.8% were CLNM (+) patients, respectively. The area under ROC was 0.837 (95% CI: 0.778–0.869).

**Conclusions:**

The risk assessment system in this study is of diagnostic value and can provide a theoretical foundation for intraoperative decision-making of prophylactic central neck dissection (pCND).

## Introduction

Thyroid carcinoma is the most common malignant endocrine tumor, of which papillary thyroid microcarcinoma (PTMC) is the most frequent pathological type. In recent years, due to the development of ultrasound (US) technology and the enhancement of people’s health awareness, the detection rate of PTMC is gradually increasing (1, 2). Studies have found that latent central lymph node metastasis (CLNM) can occur in the early stage of PTMC, especially in the central region (3), and central lymph node dissection (CLND) may increase the risk of the recurrent laryngeal nerve (RLN) and parathyroid gland injury. The rate of RLN injury after thyroid surgery is 0.3%–18.9%, and the incidences of postoperative temporary and permanent parathyroidism are 14%–60% and 4%–11%, respectively (4, 5). These complications are often the main factors of medical disputes. What is worse, the discomfort has a huge impact on the follow-up life and psychology of patients. Accurate preoperative evaluation is important to determine to operate preventive central lymph node dissection (pCND) (6).

Domestically and overseas, there is no consensus on whether to operate pCND for patients with cN0 PTMC. American Thyroid Association (ATA) Guidelines (2015 edition) (7) do not recommend pCND for cN0 PTMC patients while the domestic guidelines emphasize that the dissection can be performed prophylactically with technical support. In this study, we retrospectively analyzed the clinicopathological data of 828 cN0 PTMC patients, who underwent operation in Ningbo First Hospital, summarized the risk factors for CLNM, established a risk assessment model, and validated it, to help make a reasonable surgical plan and achieve the best treatment effectiveness.

## Methods

### Patients

The model group enrolled 500 patients who underwent thyroid operation in Ningbo First Hospital and were pathologically proved as PTMC between January 2013 and December 2015. The average age of this group was 46.8 years old. A total of 328 patients with PTMC who underwent surgery for the first time in Ningbo First Hospital from 2016 to 2017 were selected as the validation group.

Inclusion criteria: All patients were in good physical conditions before surgery, without other major diseases affecting thyroid surgery and prognosis. Preoperative US showed no suspicious signs of CLNM in patients with thyroid carcinoma. There were pathological reports after operation.

Exclusion criteria: The reports of preoperative US or fine-needle aspiration biopsy suggested CLNM. The patients had previous underlying diseases or other major diseases.

The scope of CLNM (8): upper boundary to thyroid cartilage, lower boundary to thymus, lateral boundary to the medial margin of carotid sheath, including anterior tracheal, paratracheal, and anterior laryngeal lymph nodes.

### Statistics Analysis

Software SPSS 18.0 was used for statistics analysis. *T* test was used for univariate analysis, and data were expressed as $\bar{\text{x}}+\text{s}$ . The multivariate analysis was performed with logistic regression analysis. Statistical significance was considered when *p* < 0.05.

### Risk Assessment System

The odds ratio (OR) of each independent risk factor and its 95% confidence interval could be obtained through statistical analysis. Referring to relevant literatures (9), the OR value of each independent risk factor was selected, and then assigned according to the clinical conditions. The sum was used as the risk score for CLNM. According to the risk score, the receiver operating characteristic curve (ROC) of each patient in the model group was made. The maximum value of the Yoden index was calculated to select the optimal cutoff value of the ROC curve. Meanwhile, the risk scores were stratified by Logistic regression equation, and the risk assessment system was preliminarily established. Statistical significance was considered when *p* < 0.05.

### Verifying the Risk Assessment System

#### Model Group

Hosmer–Lemeshow goodness-of-fit test was used to assess the calibration capability of the risk assessment system. The diagnostic value of the model group was evaluated in accordance with the area under the ROC curve (AUC) of model group cases. The risk score and risk degree calculated by the risk assessment system were compared with the CLNM indicated by postoperative pathology to verify its prediction reliability.

#### Validation Group

The diagnostic value of the system was verified by calculation and comparison.

## Results

### Univariate Analysis of CLNM in PTMC of Model Group

Of the 500 patients, 142 (28.4%) developed CLNM, while the remaining 358 (71.6%) did not. One-way ANOVA analysis showed that gender, tumor size, extra-glandular invasion, boundary, presence of calcification foci, accompanying blood flow, and aspect ratio >1 were independent risk factors for CLNM (*p* < 0.05). However, age, combined Hashimoto, TSH, and TPOAb had no significant relationship with CLNM (**Table 1**).

**Table 1:** Relationship between clinical features and CLNM.

Observed Factors	Amount	CLNM (-)	CLNM (+)	*p*-value
**Gender**				
Male	93	57 (61.29%)	36 (38.71%)	0.002
Female	407	301 (73.71%)	106 (26.29%)	
**Age**	500	358 (71.60%)	142 (28.40%)	
	(46.74 ± 10.86)	(47.32 ± 10.52)	(45.31 ± 11.59)	0.066
**Tumor Size (cm)**				
<0.5	195	153 (78.46%)	42 (21.54%)	0.008
≥0.5	305	205 (67.21%)	100 (32.79%)	
**Extra-glandular Invasion**				
Yes	24	10 (41.67%)	14 (58.33%)	0.002
No	476	348 (73.11%)	28 (5.88%)	
**Boundary**				
Clear	175	142 (81.14%)	33 (18.86%)	0.001
Unclear	325	216 (66.46%)	109 (33.54%)	
**Calcification**				
Yes	277	183 (66.06%)	94 (33.94%)	0.003
No	223	175 (75.11%)	48 (21.52%)	
**Multifocal**				
Yes	105	69 (65.71%)	36 (34.29%)	0.003
No	395	289 (73.16&)	106 (26.84%)	
**Aspect Ratio**				
<1	287	225 (78.40%)	62 (21.60%)	0.000
≥1	213	133 (62.44%)	80 (37.56%)	
**Blood Flow**				
Yes	275	182 (66.18%)	93 (33.45%)	0.004
No	225	176 (78.22%)	49 (21.78%)	
**Combined Hashimoto**				
Yes	97	70 (72.16%)	27 (27.84%)	1.000
No	403	288 (71.46%)	115 (28.54%)	
**TPOAb**	500	358 (71.60%)	142 (28.40%)	
	(50.1 ± 170.05)	(55.09 ± 181.86)	(37.60 ± 135.57)	0.300
**TSH**	500	358 (71.60%)	142 (28.40%)	
	(2.29 ± 2.21)	(2.31 ± 2.33)	(2.25 ± 1.89)	0.060

### Multivariate Analysis of CLNM in PTMC of the Model Group

Logistic regression was used to analyze the independent risk factors found in the above study. It was found that male (OR = 1.924, *p* = 0.011), the maximum diameter of tumor ≥ 0.5 cm (OR = 2.844, *p* = 0.037), extra-glandular invasion (OR = 3.721, *p* = 0.004), US features as unclear tumor boundary (OR = 1.674, *p* = 0.039), tumor with calcification (OR = 1.801, *p* = 0.007), and tumor aspect ratio ≥ 1 (OR = 2.056, *p* = 0.001) were independent risk factors for CLNM (**Table 2**).

**Table 2:** Independent risk factors for CLNM.

	B	S.E.	Wald	*p*-value	OR value	95% CI of OR value	
						Lower Limit	Upper Limit
Gender	0.654	0.256	6.545	0.011	1.924	1.156	3.177
Maximum Diameter of Tumor	1.045	0.501	4.357	0.037	2.844	1.066	7.590
Extra-glandular Invasion	1.312	0.458	8.190	0.004	3.721	1.512	9.116
Boundary	0.515	0.249	4.272	0.039	1.674	1.027	2.728
Calcification	0.588	0.218	7.292	0.007	1.801	1.175	2.760
Focus	0.152	0.252	0.364	0.546	1.164	0.711	1.906
Aspect Ratio	0.721	0.217	10.998	0.001	2.056	1.343	3.149
Blood Flow	0.342	0.227	2.274	0.132	1.407	0.903	2.195
Constant	−2.969	0.383	60.019	0.000	0.051		

### Establish a Risk Assessment System for CLNM in PTMC

Six independent risk factors were assigned in combination with clinical conditions (10) (**Table 3**). The sum of the scores of each risk factor was used as the risk score of CLNM in patients with PTMC. This scoring method was used to calculate the risk score of 500 patients in the model group, and the ROC curve of the risk score was made. By calculating the maximum value of the Yoden index, the critical value of the risk score for predicting the occurrence of CLNM in PTMC was 5.5 (sensitivity = 73.2%, specificity = 59.5%); thus, risk score was classified.

**Table 3:** Assignment of independent risk factors.

Independent risk factors	Score	
	Yes	No
Male	2	0
Maximum tumor diameter ≥ 0.5 cm	3	0
Extra-glandular invasion	4	0
US showed that the tumor boundary was unclear	2	0
US showed tumor with calcification	2	0
US showed that the aspect ratio of tumor was ≥ 1	2	0

To classify the risk score of CLNM in PTMC further, the logistic regression equation was established.


$$ln=\left(\frac{P}{1-P}\right)=0.654X_{1}+1.045X_{2}+1.312X_{3}+0.515X_{4}+0.588X_{5}+0.721X_{6}-2.969$$

X1: gender, male X1 = 1, female X1 = 0;

X2: maximum diameter of tumor, ≥0.5 cm X2 = 1, <0.5 cm X2 = 0;

X3: extra-glandular invasion, yes X3 = 1, no X3 = 0;

X4: boundary, unclear or not clear enough X4 = 1, clear or barely clear X4 = 0;

X5: calcification, with X5 = 1, without X5 = 0;

X6: aspect ratio, ≥1 X6 = 1, <1 X6 = 0.

**Figure 1:**
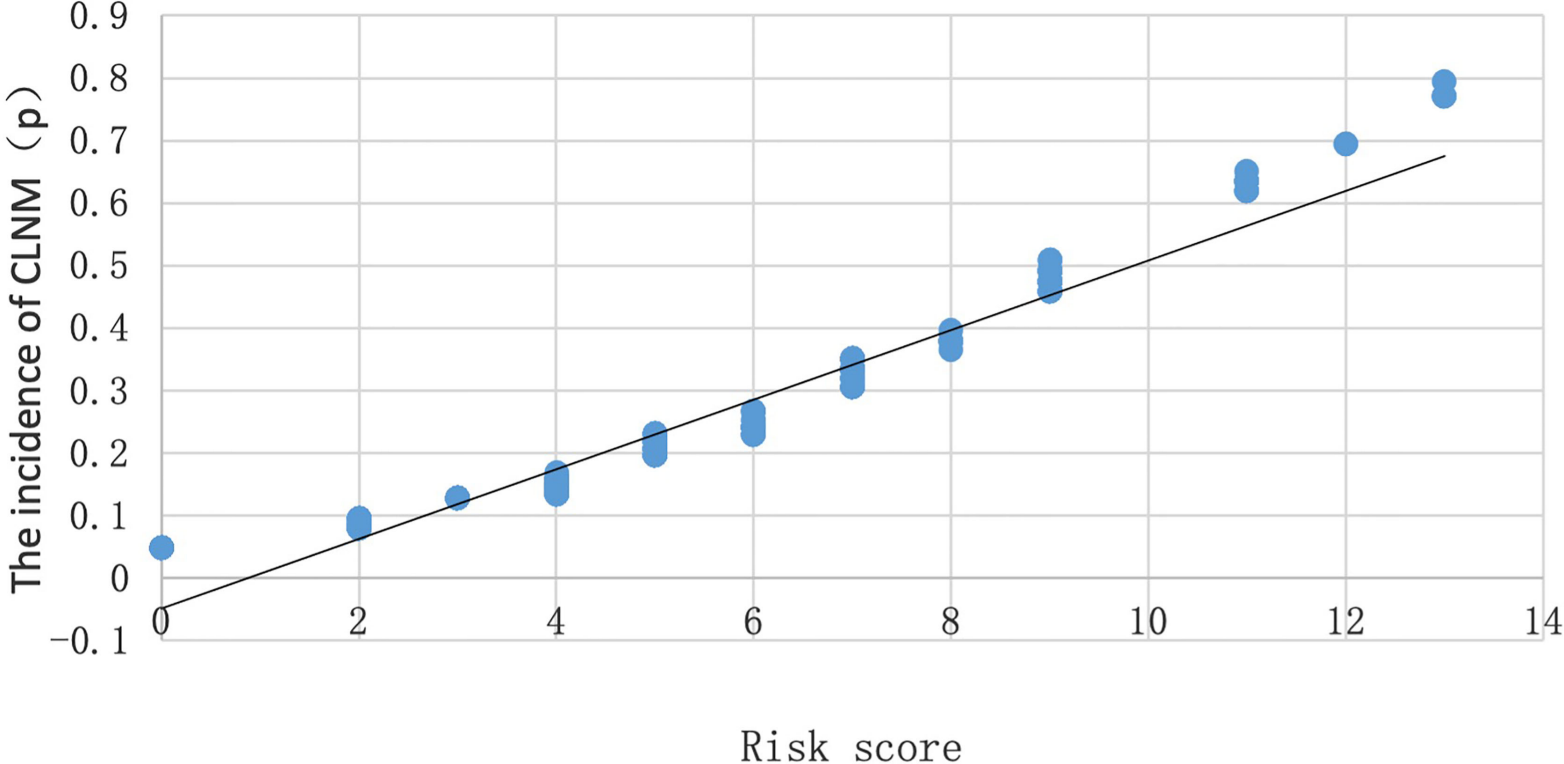
Scatter plot of the relationship between the probability of occurrence (P) and risk score of CLNM.

According to the regression equation, the incidence (P) of CLNM in PTMC of 500 cases in the model group could be calculated by substituting the specific properties into the equation. When *p* = 0.5, it was considered that the incidence of CLNM in PTMC is the same as that without CLNM. When *p* > 0.5, it was considered that the incidence of CLNM in PTMC is higher than that without CLNM. To prove the correlation between the risk scores calculated by assignment and the incidence of CLNM in PTMC calculated by regression equation, a scatter plot was made (**Figure 1**). It is found that there was a linear correlation between the incidence of CLNM in PTMC and the risk scores; the incidence of CLNM rose gradually with the increase of CLNM risk scores. As shown in the scatter plot, when the risk score is 9, *p* = 0.5.

Therefore, a risk assessment system for predicting CLNM in PTMC was preliminarily established: ≤5 was classified as low risk, 6–8 was classified as medium risk, and ≥9 was classified as high risk.

### Risk Assessment System Verification

#### Verification of the Model Group

##### Hosmer–Lemeshow Goodness-of Fit Test

Hosmer–Lemeshow goodness-of-fit test was used for multivariate analysis of the model group with logistic regression. The evaluation result was *χ* ^2^ = 3.641, *p* = 0.888, suggesting that the predicted value and the actual value had no statistical significance, indicating that the fitting degree was high, and the calibration ability was good.

##### The Evaluation of the Value of System by ROC Curve

**Figure 2:**
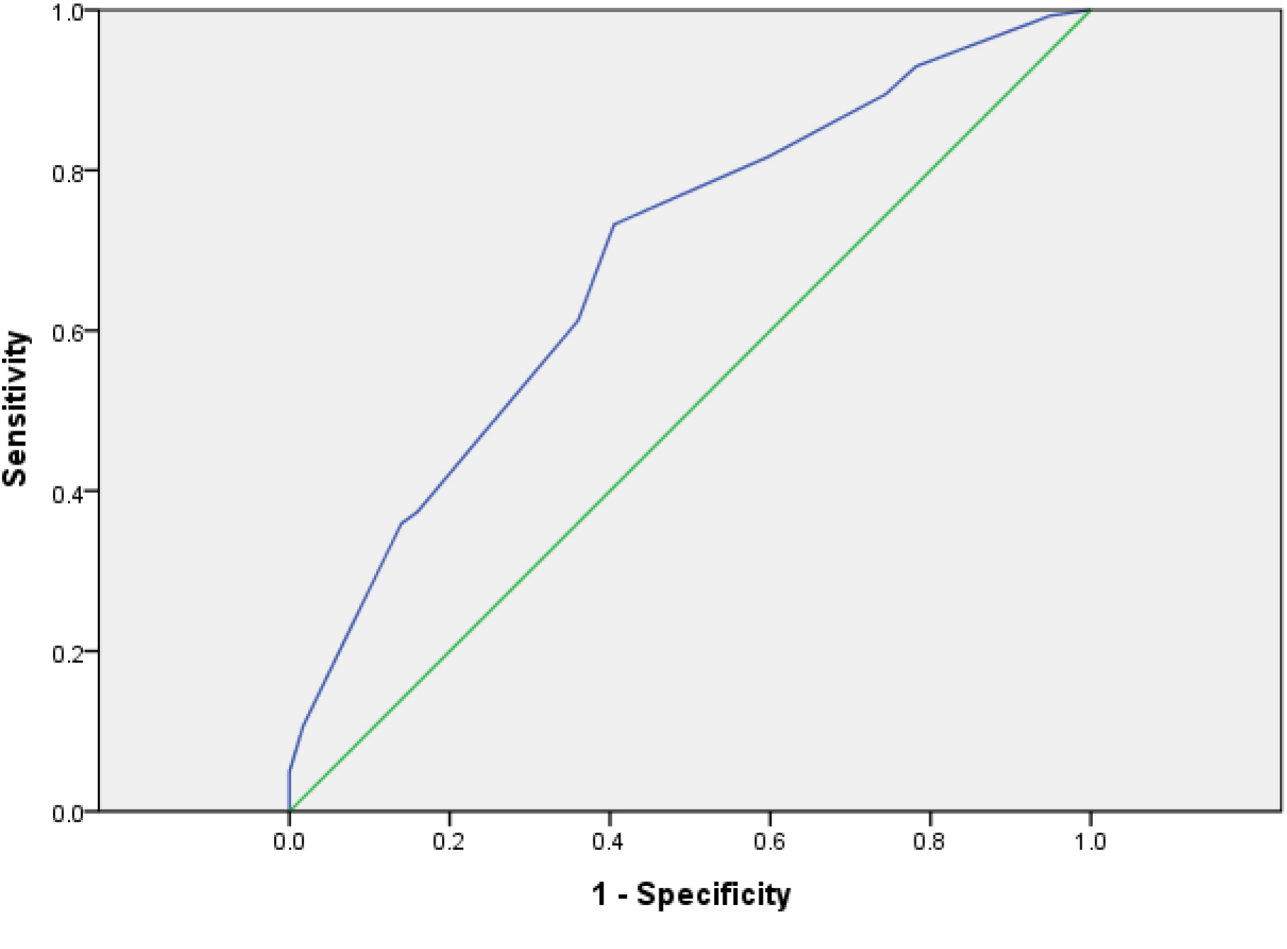
ROC curve of risk factor scores in model group.

The risk of the model group was scored with the risk assessment system. The AUC was 0.687 (95% CI: 0.635–0.783) and the cutoff was 5.5 (*p* < 0.01) (**Figure 2**). It showed that the risk assessment system had good diagnostic value.

##### Comparison and Verification With Postoperative Pathological Results

**Figure 3:**
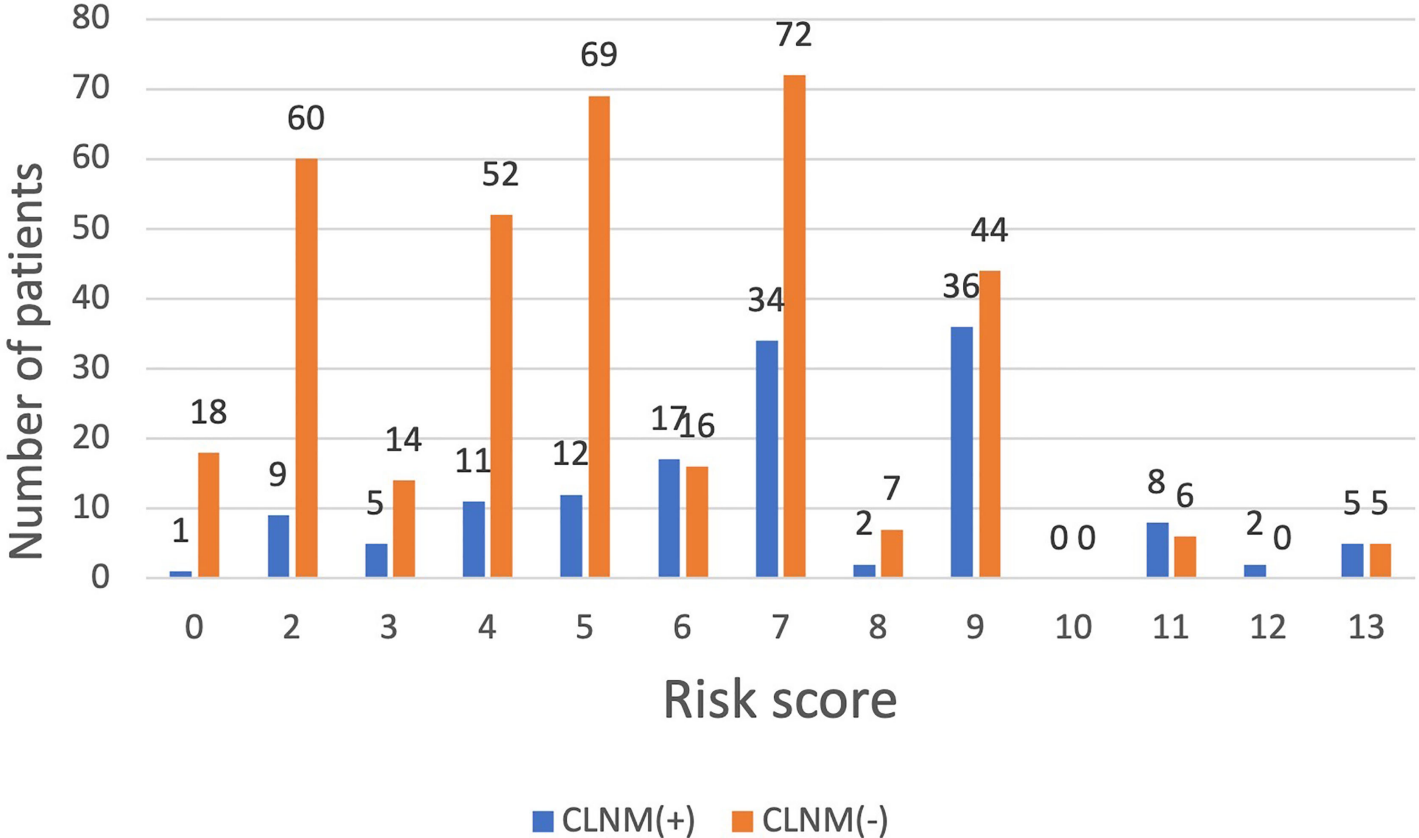
The distribution of CLNM-negative and positive patients with different risk scores inthe model group.

Among the 500 patients in the model group, the proportions of low-risk, medium-risk, and high-risk CLNM (+) patients were 15.1%, 35.8%, and 50.5%, respectively, while the proportions of low-risk, medium-risk, and high-risk CLNM (-) patients were 84.9%, 64.2%, and 49.5% respectively (**Figure 3**). According to the risk scores, there was significant difference in the distribution of low risk, medium risk, and high risk between the CLNM (+) and CLNM (-) group (*χ* ^2^ = 137.669, *p* < 0.001) (**Table 4**).

**Table 4:** Model group scoring.

CLNM	Risk Score			*p*-value
	Low risk	Medium risk	High risk	<0.001
	(≤5)	(5–8)	(≥9)	
**(-)**	213	95	50	
**(+)**	38	53	51	

#### Verification of the Validation Group

##### Establishment of Validation Group Database

From 2016 to 2017, 328 patients with PTMC were selected as the validation group, among which 106 cases were CLNM (+), accounting for 32.3%, while 222 cases were CLNM (-), accounting for 67.7%.

##### The Evaluation of the Value of System by ROC Curve

**Figure 4:**
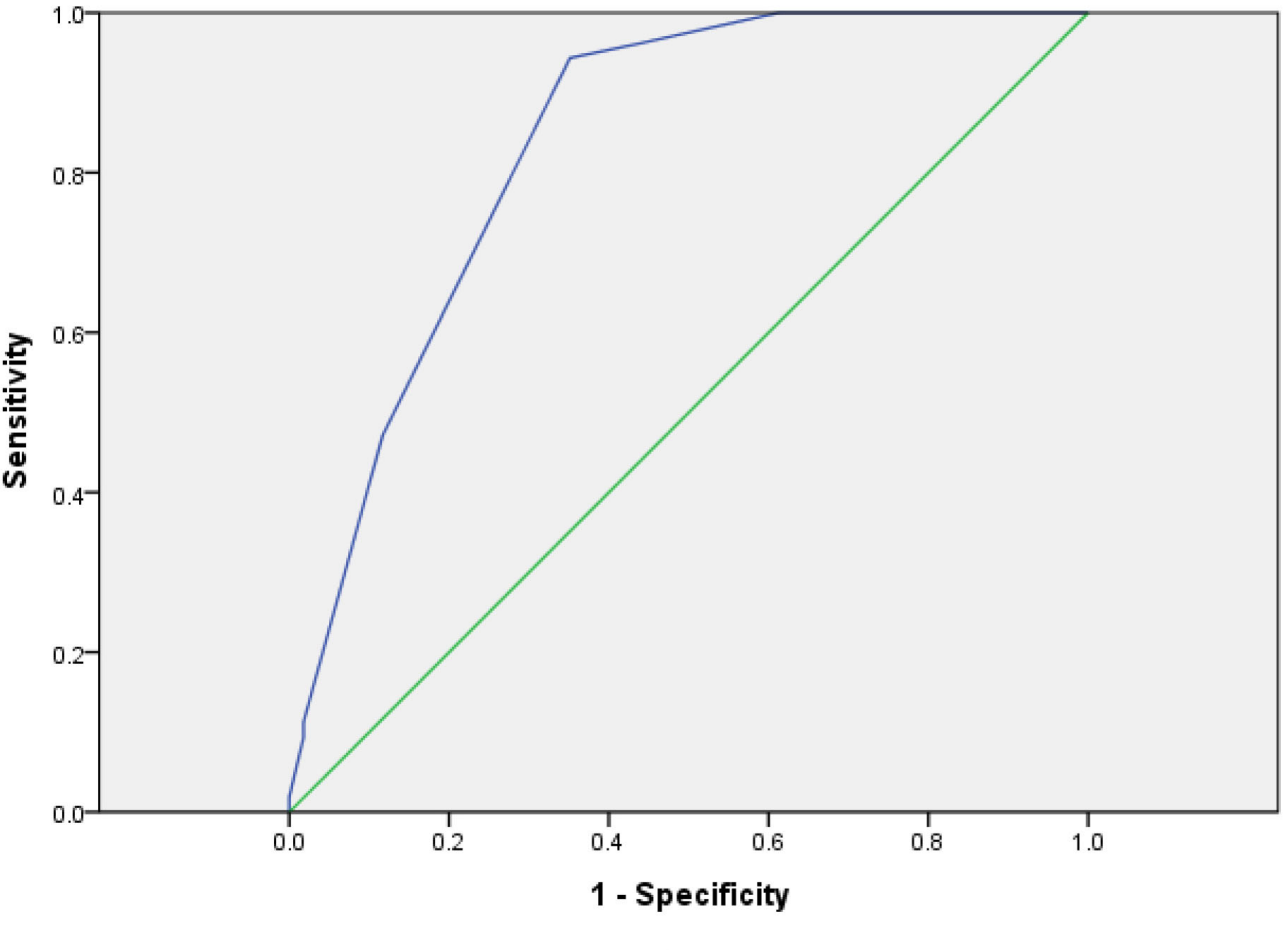
Validation group risk score ROC curve.

Risks of 328 patients were assessed with PTMC. The results of ROC showed that the cutoff value of the system was 5.5 (Sensitivity = 0.962, Specificity = 0.559, AUC = 0.837, *p* < 0.001) and the 95% CI was 0.778–0.869 (**Figure 4**), which proved that the risk assessment system had good diagnostic value.

##### Comparison and Verification With Postoperative Pathological Results

**Figure 5:**
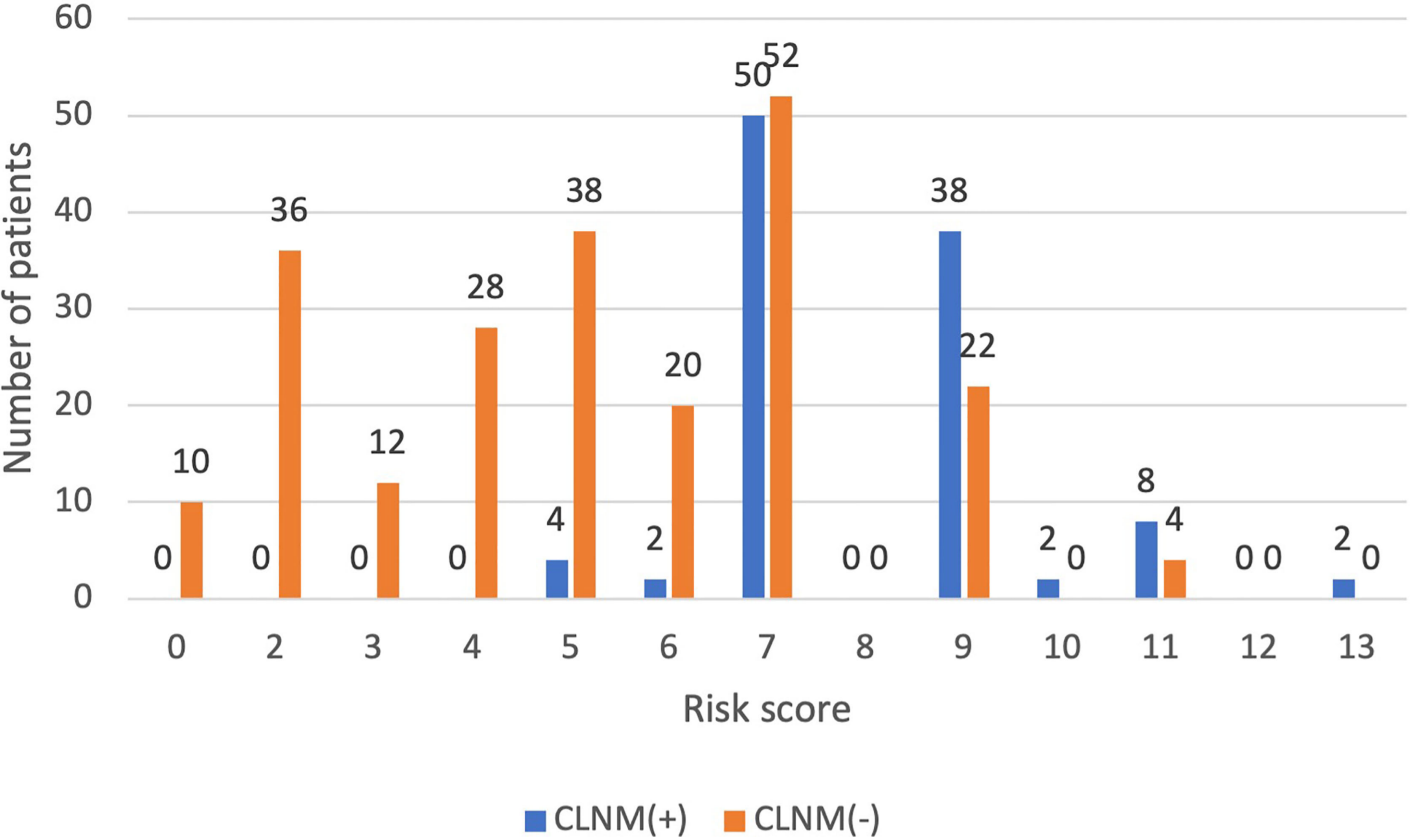
The distribution of CLNM-negative and positive patients with different risk scores inthe validation group.

Comparing the patient scores with their actual pathological results, it was found that in the low-risk group, CLNM (+) patients accounted for 3.1% and CLNM (-) patients accounted for 96.8%; in the high-risk group, CLNM (+) patients accounted for 65.8%, and CLNM (-) patients accounted for 34.2%; the proportions of CLNM (+) patients and CLNM (-) patients were 41.9% and 58.1%, respectively (**Figure 5**). That is to say, the distribution of low risk, medium risk, and high risk between CLNM (+) group and CLNM (-) had significant difference (*χ* ^2^ = 47.021, *p* < 0.001) (**Table 5**).

**Table 5:** Validation group scoring.

CLNM	Risk Score			*p*-value
	Low risk	Medium risk	High risk	<0.001
	(≤5)	(5–8)	(≥9)	
**(-)**	124	72	26	
**(+)**	4	52	50	

## Discussion

Thyroid cancer is a common malignant tumor with a 10-year survival rate of more than 90% (11). It was found that CLNM may occur to 40%–90% of PTMC patients (12–14). CLNM will increase the risk of postoperative recurrence and affect the prognosis of patients (15–17). Although thyroid surgery is one of the most common and safest procedures in endocrine surgery, the risk of complications is still unavoidable on account of the unique anatomy of the thyroid gland (18, 19). Numbness of hands and feet, difficulty in articulation, and even inability to breathe are still the major problems perplexing surgeons. These issues need to be addressed, especially in an era when changes in quality of life are incorporated into surgical outcomes (20, 21).

pCND is more prone to RLN injury because the path of the RLN is included in the surgical field of CLND.

There are many possible causes of injury to the RLN, such as traction, ligation, heat injury, and so on. Traction is the main cause of injury to the RLN (22, 23). So, how to effectively reduce traction will play an important role in the protection of RLN.

Due to the connective tissue, the RLN should be dissected from the lymph node capsule prior to CND, which could reduce the probability of injury to the RLN (22).

This was also a test of the operator’s ability. The invention of energy-based devices (EBDs) is a great progress in thyroid surgery. EBDs can decrease operative time, incision length, blood loss, pain, wound drainage, neck hematoma, and hypocalcemia (24–27). However, the heat from the EBDs can cause iatrogenic thermal injury to the RLN. A study of 11,355 patients who underwent thyroidectomy found a higher rate of hoarseness with EBDs compared to the non-EBD group (24, 28). A 10-year meta-analysis also found that the use of EBDs could cause a higher rate of paralysis (29). IONM allows real-time monitoring of RLN function through continuous intraoperative neural monitoring, significantly improving postoperative quality of life (30, 31). However, IONM is not widely used in clinical practice due to its high cost and the consumable medical supplies. At the same time, the false-positive rate of IONM, the lack of experience with IONM, and the failures of IONM instrument are often the reasons that hinder the application of IONM in clinic (32, 33).

For recurrent PTMC patients, reoperation may not only significantly increase surgical complications, but also decrease the life quality of patients. Therefore, how to deal with the cervical lymph nodes during the initial operation is particularly important.

Domestic experts have different opinions on whether to perform pCND. Some experts think pCND can fundamentally eliminate the occult metastasis lymph node in central region and reduce tumor recurrence or lymph node metastasis. More importantly, it can avoid surgical complications that may be caused by the second operation, and also reduce the financial and mental burden of patients (34, 35). However, some experts disagree to perform pCND. When lymph node metastasis occurs, treatment has no significant impact on the postoperative survival rate of patients. What is more, avoiding pCND can reduce the incidence of postoperative complications caused by surgery (36, 37).

For PTMC patients with unclear tumor boundary, tumor with calcification, and tumor aspect ratio ≥ 1, the incidence of CLNM is higher. However, it should be noted that our result depends on the operator’s diagnostic experience to a large extent, as well as the US instrument’s resolution, which may lead to an impact to research results.

Through retrospective analysis and logistic regression equation, this study pioneered a complete risk assessment system for CLNM in PTMC and determined that the score ≤ 5 is low risk, 6–8 is medium risk, and ≥9 is high risk. The area under the ROC curve of the risk score in the model group and that in the validation group were >0.5, which proved that the assessment system had high diagnostic value. Substituting pathological information of the model group and the validation group were into the risk assessment system. We found that CLNM (-) accounted for the majority of patients with low-risk scores (model group: 84.9%; validation group: 96.8%). That is, the risk of CLNM in PTMC of low-risk patients was low. Among the high-risk patients, the proportion of CLNM (+) patients was higher than that of CLNM (-) patients (model group: 50.5% vs. 49.5%; validation group: 65.8% vs. 34.2%); in other words, the risk of CLNM in PTMC in high-risk patients was relatively high. The proportion of CLNM (+) patients in medium-risk patients is slightly higher (model group: 35.8% vs. 64.2%; validation group: 41.9% vs. 58.1%). However, compared with low-risk patients, the incidence of CLNM was significantly increased. Therefore, these patients should be cautious in choosing whether to perform pCND.

There are several previous studies regarding prediction models for CLNM. Wang et al. (38) analyzed the factors of Level VI metastasis in PTMC with stage cN0. However, the sample size of this study was small and lacks further verification. Although Jiang et al. (39) analyzed 4,884 PTMC patients from 2 hospitals, they only explored related risk factors. Our model not only screened related risk factors, but also established relevant formulas through statistical analysis to furthermore conveniently assess the risk of central region lymph node metastasis in PTMC patients.

Through AUC calculation, Hosmer–Lemeshow goodness-of-fit test as well as comparison between assessment results analyses and actual pathological conditions, it is suggested that the risk assessment system had certain diagnostic value. All risk factors mentioned above can be easily obtained before CLND. Such a risk assessment system is simple and convenient for clinical application, and conducive to assisting physicians in intraoperative decision-making. The analysis shows that CLND is not recommended for low-risk patients, but for high-risk patients. As for medium-risk patients, whether to perform pCND should be considerably decided in combination with the operator’s surgical ability and the patient’s comprehensive situation. So as to realize the precise treatment to PTMC patients, which not only reduces the risk of local recurrence and distant metastasis, but also avoids the injury of recurrent laryngeal nerve and parathyroid gland caused by pCND.

At present, the current findings about the treatment of PTMC lymph nodes are not consistent at home and abroad. In this study, we retrospectively analyzed the clinicopathological data of 828 patients with cN0 PTMC, and established and verified a prediction model. For patients with PTMC in stage cN0, we recommend to perform pCND, especially for medium-risk and high-risk patients. However, we refer to this model for some patients who have underlying diseases, or who are strongly worried about postoperative thyroid complications, or who resist surgery strongly, or whose rapid frozen sections during operation suggest a suspected malignant tumor. For those low-risk patients screened by the model, we can choose not to pCND or recommend them to follow-up closely. Meanwhile, in future clinical practice, PTMC patients with stage cN0 who undergo pCND can be classified according to the model. We can verify this model by comparing the prognosis of the three risk levels.

In the next phase, we plan to carry out long-term follow-up of patients to master their postoperative survival, whether they have postoperative local recurrence, distant metastasis, or even death. In the meantime, we will improve the risk assessment system further *via* multi-center and large-sample prospective clinical research, to achieve its better clinical guidance.

## Data Availability Statement

The original contributions presented in the study are included in the article/supplementary material. Further inquiries can be directed to the corresponding author.

## Ethics Statement

The studies involving human participants were reviewed and approved by the Ethics Committee of Ningbo First Hospital. Written informed consent for participation was not required for this study in accordance with the national legislation and the institutional requirements.

## Author Contributions

JW, XS, and YG contributed to the concept design, planning of the study, revision, and final approval of the present article. JW, YD, JZ, and LS were responsible for gathering the data, writing, analysis, revision, and final approval of the present article. All authors contributed to the article and approved the submitted version.

## Funding

This study was funded by the Medical Science and Technology project of Zhejiang Province (grant no. 2022KY1110) and Key Disciplines of Ningbo First Hospital.

## Conflict of Interest

The authors declare that the research was conducted in the absence of any commercial or financial relationships that could be construed as a potential conflict of interest.

## Publisher’s Note

All claims expressed in this article are solely those of the authors and do not necessarily represent those of their affiliated organizations, or those of the publisher, the editors and the reviewers. Any product that may be evaluated in this article, or claim that may be made by its manufacturer, is not guaranteed or endorsed by the publisher.
